# *TERT* Promoter Mutation Analysis of Whole-Organ Mapping Bladder Cancers

**DOI:** 10.3390/genes12020230

**Published:** 2021-02-05

**Authors:** Veronika Weyerer, Markus Eckstein, Pamela L. Strissel, Adrian Wullweber, Fabienne Lange, Lars Tögel, Carol I. Geppert, Danijel Sikic, Helge Taubert, Sven Wach, Bernd Wullich, Arndt Hartmann, Robert Stoehr, Johannes Giedl

**Affiliations:** 1 Institute of Pathology, University Hospital Erlangen, Friedrich-Alexander Universität Erlangen-Nürnberg, 91054 Erlangen, Germany; markus.eckstein@uk-erlangen.de (M.E.); strisspa@outlook.com (P.L.S.); aw@smartandfair.de (A.W.); fabienne.lange@uk-erlangen.de (F.L.); lars.toegel@uk-erlangen.de (L.T.); carol.geppert@uk-erlangen.de (C.I.G.); arndt.hartmann@uk-erlangen.de (A.H.); robert.stoehr@uk-erlangen.de (R.S.); johannes.giedl@pathologie-weiden.de (J.G.); 2 Department of Gynecology and Obstetrics, University Hospital Erlangen, Friedrich-Alexander-Universität Erlangen-Nürnberg (FAU), 91054 Erlangen, Germany; 3 Department of Urology and Pediatric Urology, University Hospital Erlangen, Friedrich-Alexander Universität Erlangen-Nürnberg, 91054 Erlangen, Germany; danjiel.sikic@uk-erlangen.de (D.S.); helge.taubert@uk-erlangen.de (H.T.); sven.wach@uk-erlangen.de (S.W.); bernd.wullich@uk-erlangen.de (B.W.)

**Keywords:** *TERT* promoter mutation, whole-organ mapping bladder tumor, clonality

## Abstract

Background: Multifocal occurrence is a main characteristic of urothelial bladder cancer (UBC). Whether urothelial transformation is caused by monoclonal events within the urothelium, or by polyclonal unrelated events resulting in several tumor clones is still under debate. *TERT* promoter mutations are the most common somatic alteration identified in UBC. In this study, we analyzed different histological tissues from whole-organ mapping bladder cancer specimens to reveal *TERT* mutational status, as well as to discern how tumors develop. Methods: Up to 23 tissues from nine whole-organ mapping bladder tumor specimens, were tested for *TERT* promoter mutations including tumor associated normal urothelium, non-invasive urothelial lesions (hyperplasia, dysplasia, metaplasia), carcinoma in situ (CIS) and different areas of muscle invasive bladder cancers (MIBC). The mutational DNA hotspot region within the *TERT* promoter was analyzed by SNaPshot analysis including three hot spot regions (−57, −124 or −146). Telomere length was measured by the Relative Human Telomere Length Quantification qPCR Assay Kit. Results: *TERT* promoter mutations were identified in tumor associated normal urothelium as well as non-invasive urothelial lesions, CIS and MIBC. Analysis of separate regions of the MIBC showed 100% concordance of *TERT* promoter mutations within a respective whole-organ bladder specimen. Polyclonal events were observed in five out of nine whole-organ mapping bladder cancers housing tumor associated normal urothelium, non-invasive urothelial lesions and CIS where different *TERT* promoter mutations were found compared to MIBC. The remaining four whole-organ mapping bladders were monoclonal for *TERT* mutations. No significant differences of telomere length were observed. Conclusions: Examining multiple whole-organ mapping bladders we conclude that *TERT* promoter mutations may be an early step in bladder cancer carcinogenesis as supported by *TERT* mutations detected in tumor associated normal urothelium as well as non-invasive urothelial lesions. Since mutated *TERT* promoter regions within non-invasive urothelial lesions are not sufficient alone for the establishment of cancerous growth, this points to the contribution of other gene mutations as a requirement for tumor development.

## 1. Introduction 

Telomerase reverse transcriptase (*TERT*) promoter mutations occur in 60–80% of all urothelial bladder cancers (UBC) independent of tumor stage and grading thus, represent the most frequent alteration in this tumor entity [1]. With each cell cycle during DNA replication under physiological conditions, a loss of DNA occurs at chromosomal telomeres, however the length is regulated through *TERT* [2]. DNA mutations occurring in the core promoter region cause telomerase aberrant activation and lead to unlimited cellular proliferation [3]. *TERT* promoter mutations in UBC are found in 99% of tissue samples at position −124 and −146 base pairs upstream from the ATG transcriptional start site position and are responsible for aberrant telomerase activity [1]. Moreover, due to high *TERT* mutational incidence rates in UBC several studies proposed a possible role implementing these alterations in urinary testing and as a follow-up diagnostic tool [4].

UBC generally presents as a multifocal tumor with either simultaneous or metachronous developed tumors. However, it is still unclear if urothelial transformation is caused by monoclonal events, leading to identical tumor foci or by polyclonal independent events. Different explanations have been proposed how these transformation events occur. One theory incorporates the idea of monoclonal cells migrating through different tissue layers as well as in regions of the bladder wall. On the other hand, tumor cells could be seeded intraluminally through the urine and thereby a second implantation site initiated. Furthermore, the influence of carcinogens floating in the urine can affect the bladder wall and thereby initiate different tumor clones causing a field cancerization [5].

To unravel these different theories of UBC development we tested if *TERT* promoter mutations occur early in proposed pre-stage tissues associated with the tumor and play a role during tumorigenesis. Therefore, we analyzed *TERT* mutations at different known promoter nucleotide positions using a large cohort of whole-organ mapping bladder tumors. 

## 2. Materials and Methods

### 2.1. Whole-Organ Mapping Bladder Tumor Specimens and Strategy

**Figure 1:**
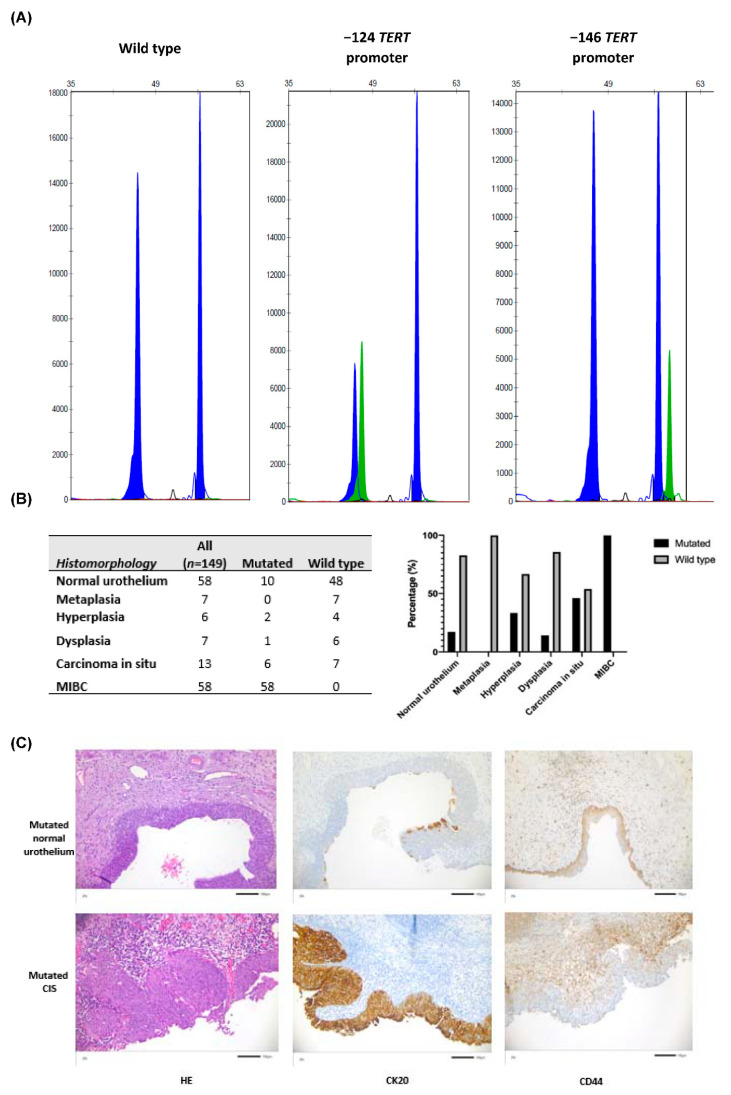
(**A**) Representative SNaPshot analysis of the *TERT* promoter hot spot mutations. The X-axis represents base pair length of the DNA fragment and the Y-axis represents the intensity of fluorescence signal of the labeled nucleotide. The Blue peak for each promoter mutations corresponds to a Guanine and the green peak an Adenine. (**B**) Total numbers as well as percentages of *TERT* mutated and wild type sequences analyzed within each tissue group are shown. (**C**) Representative Hematoxylin and Eosin, CK20 and CD44 stained images of *TERT* promoter mutated tumor associated normal urothelium and *TERT* promoter mutated CIS (all magnification: 200×). *TERT* mutated tumor associated urothelium demonstrated the physiological expression of CK20 staining within the umbrella cells and CD44 expression in the basal and over lying tissue layers. In contrast, in *TERT* mutated CIS, CK20 was abnormally expressed not only in the umbrella cells but also in adjacent tissue layers, and expression of CD44 was restricted to the basal layer.

**Figure 2:**
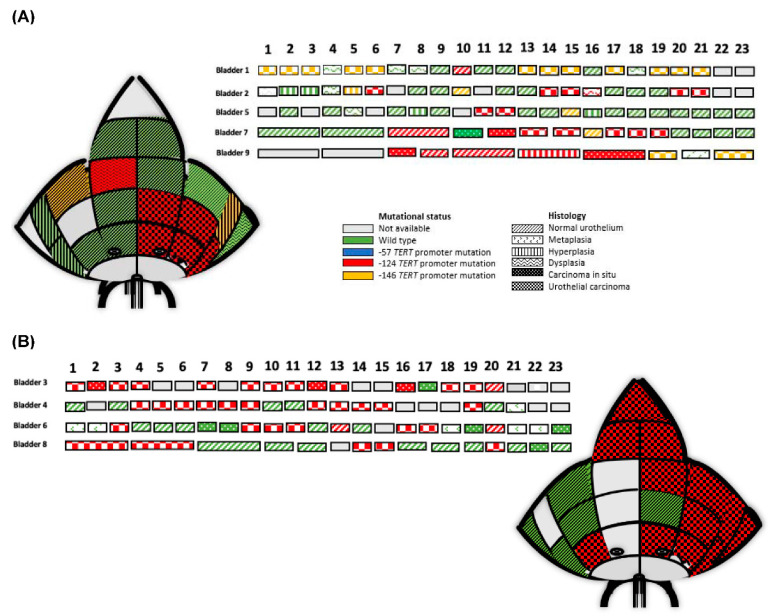
Two representative whole organ mapping bladder schematic diagrams are shown for Bladder 2 (left above) and Bladder 4 (right below), which demonstrate the overall orientation of 23 different tissue histologies, their macroscopic positions as well as the specific *TERT* promoter mutations. In addition, all nine whole organ mapping bladder specimens (Bladder 1–9) and their respective 23 different tissue histologies, *TERT* promoter mutational status and indicated tissue macroscopic positions are illustrated as single rectangles in rows. For Bladder 7, 8 and 9 some areas could only be represented as one larger region as illustrated within the figure. (**A**) Five polyclonal bladder cancer specimens demonstrating different tissue histologies as well as the *TERT* promoter mutational status. (**B**) Four monoclonal whole-organ mapping bladder cancer specimens demonstrate different tissue histologies and *TERT* promoter mutational status.

Archival material of the Institute of Pathology, Erlangen was retrospectively evaluated and available bladder cancer specimens diagnosed as MIBC were screened for *TERT* promoter mutations. This analysis resulted in a cohort of nine whole-organ bladder tumor specimens with identified *TERT* promoter mutations in the carcinoma, which could then be further evaluated. All MIBC were derived from a complete cystectomy and opened with a Y-shaped incision for further examination. For a single whole-organ mapping bladder specimen twenty-three defined regions were dissected macroscopically where each region could potentially house distinct proposed pre-stage tissues including tumor associated normal urothelium as well as non-invasive urothelial lesions (hyperplasia, dysplasia, metaplasia) and CIS as well as the tumor mass [6]. All these different tissues are routinely banked at the Institute of Pathology, Erlangen. Defining all tissue histologies in relation to each other one can create an entire map within a whole-organ bladder tumor specimen. This model system is powerful model to study tumorigenesis as demonstrated previously [7]. A schematic of tissue sampling is presented in Figure S1. All tissues used in this study were pathologically re-evaluated by two Uropathologists (V.W., A.H.) according to the latest TNM staging manual of the UICC (8th edition, 2017) and the WHO 2016 classification for tumors of the genitourinary tract [8]. Pathological and clinical characteristics as well as identification numbers of each whole organ bladder tumor (named Bladder 1–9) are presented in Table 1 and Figure 1. For Bladder 7, 8 and 9 sampling of defined tissue positions varied from the schematic with changes illustrated in Figure 2. Histologies from all nine whole organ specimens are also shown in detail in Figure 2. For further analysis, using immunohistochemistry as well as DNA isolation, consecutive tissue cuts from each tissue block containing the pathological tissue were prepared. This study was conducted in accordance with the Declaration of Helsinki, and the protocol approved by the Ethics Committee of the Friedrich-Alexander University Erlangen-Nürnberg (No. 329_16B). 

### 2.2. Immunohistochemical Analysis of Tumor Associated Normal Urothelium and Non-Invasive Urothelial Lesions from Whole-Organ Mapping Bladder Cancer Specimens

All *TERT* promoter mutated tumor associated normal urothelium as well as non-invasive urothelial lesions from the whole-organ mapping bladder specimens were immunohistochemically evaluated for CK20, CD44, TP53 and MIB1 staining. CK20 and CD44 were chosen as differentiation markers, which show distinct staining patterns among normal urothelium as well as for non-invasive urothelial lesions thus, are used for routine diagnostic evaluation. P53 evaluation as well as MIB1 staining is also used for evaluation of distinguishing normal urothelium as well as for non-invasive urothelial lesions [8]. Whole tissue consecutive cuts were made from embedded tissues containing *TERT* promoter mutations and used for immunohistochemistry as preformed with a Ventana BenchMark Ultra (Ventana, Indianapolis, IN, USA) and a Dako Link 48 (Dako, Santa Clara, CA, USA) autostainer accreditated by the German Accreditation Office (DAKKs) according to DIN EN ISO/IEC 17020. Detail information of the used antibodies are displayed in Table 2. 

### 2.3. DNA Isolation 

The tissue component from each tumor associated normal urothelium as well as non-invasive urothelial lesions, CIS and MIBC was manually microdissected from marked areas on each consecutive tissue slide derived from its corresponding tissue block in order to achieve at least 80% purity. DNA isolation was performed using the DNA preparation kit (Maxwell^®^ 16 System, Promega, Mannheim, Germany) according to the manufacturer’s instructions. 

### 2.4. TERT Promoter Mutation Analysis 

Mutation analysis of the *TERT* gene promoter was performed as previously described [6]. Due to different amounts of DNA as well as degradation from formalin-fixed and paraffin-embedded microdissected tissues might be expected and that *TERT* promoter mutations are mainly identified at hot spot regions, we used the established, highly sensitive, low-cost SNaPshot analysis based on the published model by Hurst et al. in 2014 [9]. We implemented the previously reported SNaPshot assay (Life Technologies Corp, Carlsbad, CA, USA) for the detection of the three hotspot mutations located upstream at −57, −124 and −146 base pairs from the ATG transcriptional start site of the *TERT* gene. Briefly, to amplify the promoter fragment one multiplex PCR containing the −57-site and a second for the −124 and −146 sites, including the reagents and thermocycler conditions, were used as illustrated in Supplementary Table S1. Two different primer-mixes consisting of two different primers sets each (−57 forward (5′-agcacctcgcggtagtgg-3′) and −57 reverse (5′-agcccctccccttccttt-3′) or −124/−146 forward (5′-cagcgctgcctgaaactc-3′) and −124/−146 reverse (5′-gtcctgccccttcacctt-3′)) were implemented in the PCR. 

The digestion of the remaining primers and free deoxynucleotides after PCR amplification was performed with alkaline phosphatase (FastAP Thermosensitive Alkaline Phosphatase; 1U/μL; Life Technologies GmbH; Darmstadt, Germany) and an exonuclease (Exonuclease I; 20 U/μL; Life Technologies GmbH; Darmstadt, Germany). (Supplementary Table S1). The multiplex, single base primer extension PCR was performed by using the ABI PRISM^®^ SNaPshot™ Multiplex Kit (Applied Biosystems GmbH; Darmstadt, Germany). The usage of labelled dideoxynucleotides enables the identification of the nucleotide base at the site of interest (Supplementary Table S1). Two different primer-mixes consisting of different primers (-57 (5′-t(29)tcctcgcggcgcgagtttc-3′) or −124 (5′-t(19)ggggctgggagggcccgga-3′) and -146 (5′-t(34)ggctgggccggggacccgg-3′)) were used. A second digestion was performed by adding 1 μL Fast AP and using the same thermocycler conditions as illustrated in Supplementary Table S1. For the detection 0.5 μL of the sample and 19.5 μL HiDi with 0.2 μL Liz standard (GeneScan™ 120 LIZ™ dye Size Standard; Applied Biosystems GmbH; Darmstadt, Germany) were pipetted on a MicroAmp^®^ Optical 96-Well Reaction Plate (Life Technologies GmbH; Darmstadt, Germany). After a denaturation step at 90 °C for five minutes the detection was performed with capillary electrophoresis using an ABI 3500 Genetic analyzer (Applied Biosystems GmbH; Darmstadt, Germany).

### 2.5. Telomere Length Determination

Telomere length was analyzed by Relative Human Telomere Length Quantification qPCR Assay Kit (ScienCell, Carlsbad, CA, USA) according to the Manufacturer’s instruction. Telomere length is recognized and amplified by comparing samples to reference genomic DNA containing a 100-base pair (bp) telomere sequence located on human chromosome 17. The total as well as the average telomere length was then calculated. 

### 2.6. Statistical Analysis

Descriptive statistical analysis was used to characterize the nominal variables in terms of frequency and percentages. A non-parametric Wilcoxon rank-sum test was used for comparison between continuous variables. All analysis was performed by GraphPad Prism 7.2 (GraphPad Software Inc., San Diego, CA, USA) and JMP SAS 13.4 (SAS). *p*-Values < 0.05 represented statistical significance.

## 3. Results 

### 3.1. TERT Promoter Mutations Were Identified within Tumor Associated Normal Urothelium, Non-Invasive Urothelial Lesions, CIS and MIBC from Whole-Organ Mapping Bladder Cancer Specimens

From 149 available tissue samples, 75 (50.33%) *TERT* promoter mutated regions were identified. Figure 1A illustrates representative sequence results of the SNaPshot assay. Among all positions there were no −57 hot spot mutations detected. Importantly, 57 (76%) tissues were mutated at position −124 and 18 (24%) at position −146 upstream from the ATG site. Figure 1B summarizes the numbers and percentages of *TERT* promoter mutations identified among the different tissue regions within the whole-organ mapping bladder tumor specimens. Results showed that the percentages of mutated samples generally increased in a step-wise manner from 17.24% among tumor associated normal urothelium, 33.3% in hyperplasia, 14.3% in dysplasia to 46.1% of CIS specimens and 100% of all MIBC regions. Representative images of mutated tumor associated normal urothelium as well as CIS are shown in Figure 1C). 

### 3.2. Clonality and TERT Promoter Mutations

One objective of this study was to test for clonality events of *TERT* promoter mutations within the whole-organ mapping bladder cancer specimens. As shown in Figure 2 of the nine specimens, five MIBC showed a polyclonal mutational status where three MIBC presented with the −124 hot spot mutation and two MIBC with the −143 mutation. In contrast to the tumor, tumor associated normal urothelium was a different *TERT* promoter mutation compared to the MIBC. Interestingly, in Bladder #9 the CIS housed a TERT −124 mutation, which was also different to the MIBC (−146). Detailed information of the tissue histologies as well as the mutational status for the *TERT* promoter for the polyclonal events are displayed in Figure 2A. Monoclonal whole-organ mapping bladder cancer specimens are illustrated in Figure 2B, where a −124 mutation was found in MIBC, CIS and tumor associated normal urothelium. In summary, for both scenarios the MIBC always presented with the same hot spot mutation within its respective whole-organ bladder specimen and every MIBC sample was mutated. In contrast and in terms of polyclonal events, tumor associated urothelium, non-invasive urothelium lesions and CIS demonstrated a different *TERT* promoter mutation compared to the MIBC.

### 3.3. Telomere Length Analysis within the Whole-Organ Mapping Bladder Specimens

**Figure 3:**
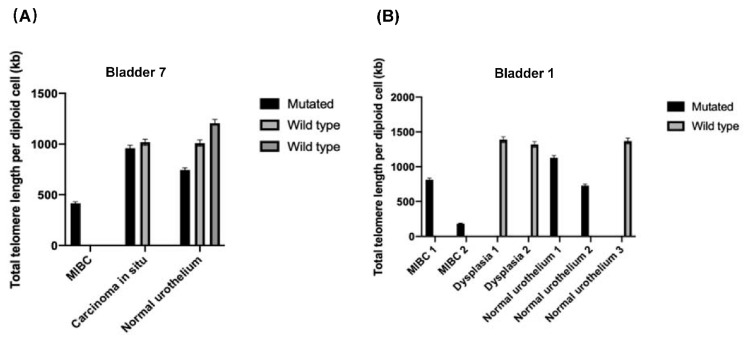
(**A**) Bladder 7 tissue specimens from the whole-organ mapping bladder specimen and amount of the total telomere length. (**B**) Same presentation for Bladder 1.

To determine if there was an association between *TERT* promoter mutations and total telomere length, some *TERT* mutated and wild type tissues as well as *TERT* mutated MIBC samples were analyzed. None of the determined telomere lengths were significantly different between mutated and wild type tissue samples (data not shown). Figure 3 presents the statistical means and standard deviations of specific histological tissues from two whole-organ bladder cancer specimens.

## 4. Discussion

In this study, we evaluated the role of *TERT* promoter gene mutations throughout nine whole-organ mapping bladder cancer specimens thus, representing a full spectrum of tumorigenesis. Our results demonstrate that adjacent and non-adjacent tumor associated urothelium, non-invasive urothelium lesions as well as CIS surrounding the tumors are *TERT* mutated. Additionally, detection of mono- as well as polyclonal mutated specimens with identification of one or several *TERT* promoter mutations strengthen both clonality hypotheses. 

*TERT* promoter mutations have been identified in the vast majority of bladder tumors independent of pathological characteristics. The hot spot mutations detected in UBC and identified among this cohort locate at −57, −124 and −146 base pairs upstream from the ATG site of the *TERT* gene and generate novel transcription factor binding sites. Similar to the first descriptions of these mutations by Allory et al. the hot spot mutation at the −124 nucleotide position was the most frequent substitution identified in our whole-organ mapping bladder cancer cohort [1]. 

Evolution of especially epithelial cancers can be demonstrated by identifying distinct histologies including dysplasia or CIS sharing both mutational backgrounds with the tumor [5,10]. Due to the anatomical site and structure, bladder cancer specimens and tumor progression of different histological tissues have been analyzed throughout an entire bladder [5,7,11]. With Smoking being the most important risk factor, the proposed induced DNA damage from carcinogens within the urine or blood stream led to the idea of field cancerization but also DNA mutations occurring in non-malignant urothelium [5,12]. This observation was recently reported by Hayashi et al. [13] identifying *TERT* promoter mutations in systematically collected normal urotheliums locating adjacent to non-invasive bladder tumor tissue. Additionally, even when the tumor was not mutated the associated normal urothelium showed a *TERT* promoter mutation. Moreover, if *TERT* mutations were initially observed, positive associations with bladder recurrence after therapy were shown indicating a potential use of *TERT* promoter gene mutations as a biomarker [13]. In line with the above findings, in this present study we also identified specific *TERT* promoter mutations in tumor associated normal urothelium but also in non-invasive urothelial lesions adjacent to or non-adjacent to muscle invasive tumors. Considering *TERT* mutations in bladder tumors, it is interesting to note that we found in contrast to non-invasive tumors described above, all MIBC tissues presented with a specific mutation within a whole-organ mapping bladder specimen. Additionally, this observation also strengthens the fact that *TERT* promoter mutations seem to be an early and crucial event during bladder tumorigenesis and importantly are independent of pathological, histological and clinical characteristics [1,14,15]. 

Clonality is widely discussed regarding bladder tumorigenesis with poly- as well as monoclonal observations. In detail, whether the process of tumor formation is due to monoclonal events within the urothelium spreading through the bladder wall or by polyclonal, events resulting in several independent tumor clones is still under debate. Findings for both theories exist and with recent advances in molecular subtyping multifocal tumors and tumor heterogeneity will even be more important in terms of planning neoadjuvant treatment regimens for patients [5]. We demonstrate in our study, that in five out of nine whole-organ mapping bladder specimen’s two hot spot mutations of the *TERT* promoter gene were identified. Interestingly, all MIBC samples within its respective bladder specimen showed the same hot spot mutation. However, in contrast to MIBC polyclonal *TERT* mutations within the same bladder specimens were identified in tumor associated normal urothelium and non-invasive urothelial lesions. This finding supports the widely accepted idea that carcinogens in the urine could damage the urothelial layer and therefore mutational backgrounds could differ. On the other hand, four out of nine analyzed whole-organ mapping bladder tumors were monoclonal for *TERT* promoter mutations pointing to the fact of a possible seeding or migration of the cells [7]. To which extent polyclonal events are influenced from *TERT* promoter mutations and how they affect follow-up diagnostic tools has to be investigated in the future [14].

The normal function of telomerase encoded by the *TERT* gene is to maintain and protect the ends of human chromosomes however, as we age they become shorter [16]. With approximately 70% of UBC harboring a *TERT* promoter mutation, functional investigations are still ongoing. In the study by Borah et al. [17] the authors investigated the complex associations of *TERT* mutated as well as wild type urothelial cell lines and observed an increased mRNA level of *TERT* transcripts, however neither the protein level nor the telomere length showed significant differences thus supporting non-translated mRNA. Additionally, Allory et al. [1] observed among 60 UBC samples no significant differences in the RNA levels of *TERT* between mutation carriers and wild types. These observations described above are comparable with our findings where there was no differences in telomere lengths. One further explanation could be that activation of the telomerase via mutations of the *TERT* promoter could also lead to other functions independent of telomere lengthening. These independent functions could affect many biological processes, including cell survival and apoptosis, DNA damage repair, mitochondrial function and stem cell activity. In addition, evidence exists that activating telomerase could also enable cells to acquire tumor-initiating mutations [3]. How *TERT* promoter mutations ultimately affect the urothelial tumor cells and additionally, implementing *TERT* promoter mutations as a diagnostic tool needs further investigation. Moreover, it was recently shown that cell lines from solid tumors with somatic *TERT* promoter mutations showed a significantly shorter telomere length compared to cell lines with a wild type *TERT* promoter [18]. Although not significant, this is in line with our findings of shortest telomere length in MIBC with *TERT* promoter mutations compared to tumor associated urothelium and non-invasive urothelium as well as CIS possibly indicating a complex interplay between *TERT* mutational activation, telomere length variation and other cellular processes. 

Limitations of our study is the retrospective nature as well as the limited, partly heterogenous sampling of the bladder cancer specimens. In addition, only hot spot mutations of the *TERT* promoter gene have been analyzed and thereby other *TERT* promoter alterations could have been missed. 

## 5. Conclusions

To our knowledge, we demonstrate for the first time in tissues from whole-organ mapping bladder tumor specimens containing MIBC, *TERT* promoter gene mutations occur in tumor associated urothelium, non-invasive urothelial lesions and CIS thus, highlighting a crucial and important role of the *TERT* gene in the development of bladder tumors. Moreover, the evaluation of distinct promoter mutant positions strengthens both theories of a mono- as well as a polyclonal development of bladder tumors. 

## Figures and Tables

**Table 1:** Study characteristics of the whole-organ mapping bladder cancer specimens.

Mapping Sample	Gender	Stage	L	V	Pn	WHO Grading 2016	WHO Grading 1973	Resection Margin	Number of Positions
Bladder 1	Male	pT3	L1	V0	P1	High-grade	G3	R0	21
Bladder 2	Female	pT3	L1	V0	Pn0	High-grade	G3	R0	19
Bladder 3	Male	pT4	L1	V1	Pn0	High-grade	G3	R0	17
Bladder 4	Female	pT3	L1	V1	Pn1	High-grade	G3	R0	18
Bladder 5	Male	pT3	L0	V1	Pn0	High-grade	G3	R0	19
Bladder 6	Female	pT3	L1	V0	Pn0	High-grade	G3	R0	22
Bladder 7	Male	pT2	L0	V0	Pn1	High-grade	G3	R0	15
Bladder 8	Male	pT3	L1	V1	Pn1	High-grade	G3	R2	14
Bladder 9	Female	pT4	L1	V1	Pn1	High-grade	G3	R1	8

WHO: World Health Organization, L: lymphovascular invasion, V: vessel invasion, Pn: Perineural invasion.

**Table 2:** Detailed information of the antibodies used in this study.

Antibody	Company	Clone	Dilution
CD44	Dako	DF1485	1:40
CK20	Dako	Ks20.8	1:50
P53	Dako	DO-7	1:50
Ki-67	Dako	MIB-1	1:100

## Data Availability

Data is contained within this article and the supplementary materials.

## Supplementary Materials

The following are available online at https://www.mdpi.com/2073-4425/12/2/230/s1, Figure S1: Different positions are collected as demonstrated, Table S1: Detail information of SNaPshot method.
