# Supplementary material for: TERT Promoter Mutation Analysis of Whole-Organ Mapping Bladder Cancers
Source: Genes (Basel). 2021 Feb 5;12(2):230. doi: 10.3390/genes12020230 (PMC7915609; doi:10.3390/genes12020230)
Supplement: Supplementary file 1 [file genes-12-00230-s001.zip › Supplementary Figure 1.pptx]

## Slide 1
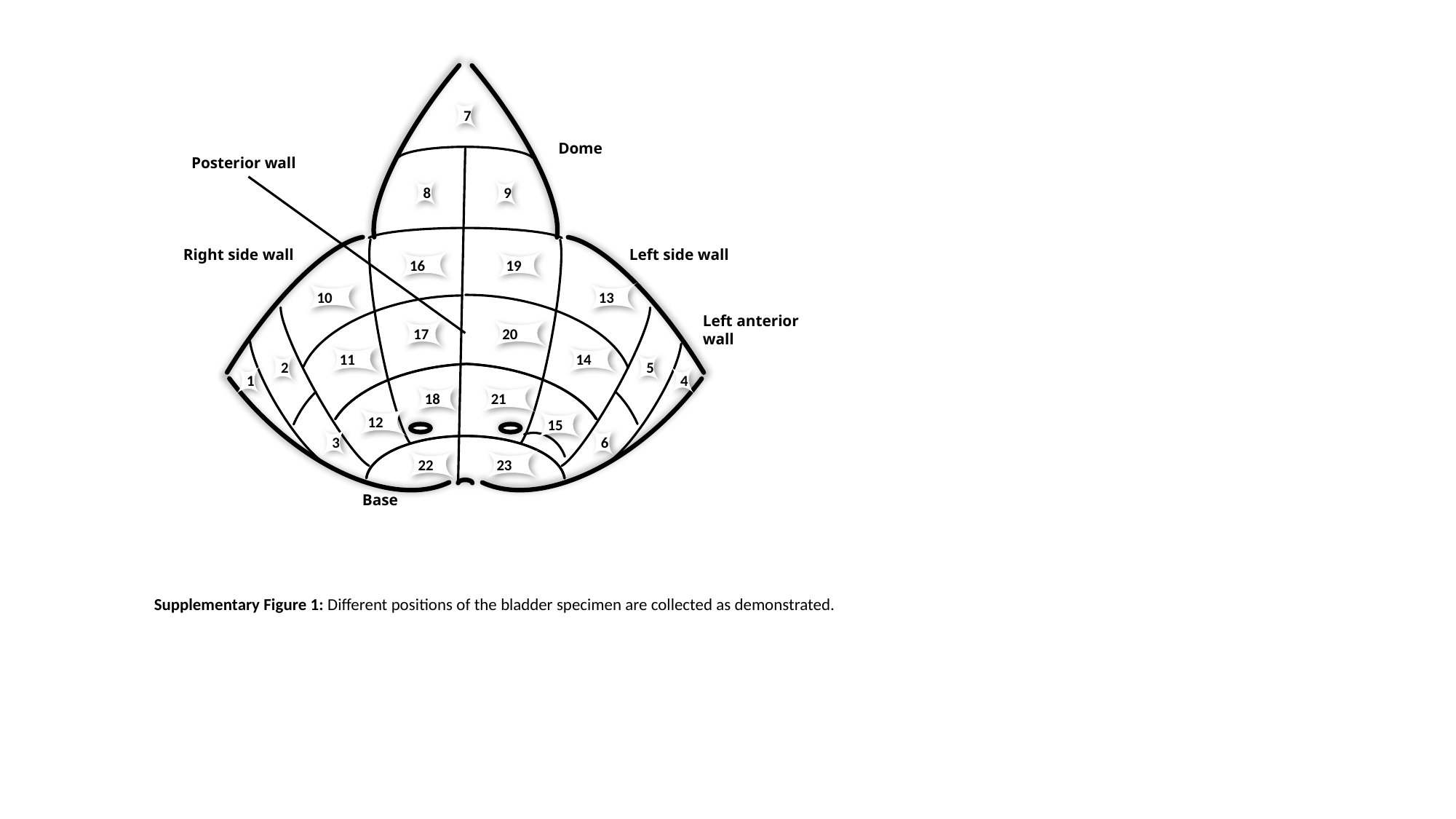

7
Dome
Posterior wall
8
9
Right side wall
Left side wall
16
19
10
13
Left anterior wall
17
20
11
14
2
5
1
4
18
21
12
15
3
6
22
23
Base
Supplementary Figure 1: Different positions of the bladder specimen are collected as demonstrated.
